# Utilization of *Scenedesmus obliquus* Protein as a Replacement of the Commercially Available Fish Meal Under an Algal Refinery Approach

**DOI:** 10.3389/fmicb.2019.02114

**Published:** 2019-09-18

**Authors:** Reeza Patnaik, Naveen Kumar Singh, Sourav Kumar Bagchi, Pavuluri Srinivasa Rao, Nirupama Mallick

**Affiliations:** Agricultural and Food Engineering Department, Indian Institute of Technology Kharagpur, Kharagpur, India

**Keywords:** algal refinery, biodiesel, bioethanol, omega-3-fatty acids, protein-rich algae meal

## Abstract

The approach of algal refinery as a method to reduce the cost of algal biodiesel by co-production of various value-added chemicals is the most up-coming strategy suggested for the economic viability of microalgal biodiesel. This concept being relatively new and novel, abundant literature on the subject is not available although fragmented data on some feedstocks are present. The main objective of this research paper is to propose an algal refinery design through utilization of *Scenedesmus obliquus* biomass for production of various industrially important products. For this purpose, first a protocol was standardized for maximum extraction of protein from *S. obliquus* biomass. Then, different experiments were conducted for 90 days each to find out the optimum concentration of microalgal protein that can be substituted in the diets of freshwater fishes for their maximum growth. During these experiments eight different growth parameters and seven water quality parameters were tested. Results showed that the standard + whole microalgal biomass + extracted microalgal protein diet (25:25:50) was the best diet for maximum growth of the freshwater fishes. After conducting these experiments, a detailed sequential extraction process for maximum valorization of the *S. obliquus* biomass or in other words an algal refinery was designed. The detailed sequential process developed, yielded 0.06 g of β-carotene, 10 g of protein, 38 g (43 mL) of biodiesel, 2 g of omega-3 fatty acid, 3 g (2.4 mL) of glycerol and 18 g (23 mL) of bioethanol from 1 Kg wet (≈100 g dry) *S. obliquus* biomass thus converting 70% of the test microalgal biomass into biodiesel and other value-added products by using an algal refinery approach.

## Introduction

The use of microalgal lipids for biodiesel production is a widely practiced strategy these days for countering the problem of over-exploitation of non-renewable sources of energy. But the production process for microalgae-derived biodiesel is extremely cost-intensive (Delrue et al., 2012; Borowitzka, 2013; Banerjee et al., 2016). Hence, application of algal refinery approach is being explored and looked upon as a possible strategy for improving the economics of microalgal biodiesel. This approach suggests the valorization of the whole microalgal biomass through production of industrially important co-products along with biodiesel for countering the costs incurred with more earnings from high-value products.

In the paper published by Patnaik and Mallick (2015), the green microalga *Scenedesmus obliquus* was used as a model organism to produce biodiesel and other industrially important co-products such as β-carotene, omega-3 fatty acids, glycerol and bioethanol in a sequential process in the designed algal refinery, but the use of protein present in the same microalgal biomass was not shown. Hence in this research study, for improved valorization of the *S. obliquus* biomass, the utilization of the leftover microalgal protein has been demonstrated by using the protein-rich algae meal as a replacement of the commercially available fish meal in the diets of the freshwater fishes.

Fishmeal is a widely used source of protein in the fish feeds. But the continued consumption of this commercially available fish meal in large quantities has reduced the availability of this protein-rich resource (Grammes et al., 2013; Jones et al., 2014; Camacho-Rodríguez et al., 2017). Hence, it is essential to find a sustainable substitute for the commercially available fish meal without affecting the quality of the feeds. Although conventional terrestrial crops such as oilseeds and grains have been successfully used as alternatives to fishmeal their effect on the nutritional quality of the fishes due to lack of certain essential amino acids in the terrestrial crops (Valente et al., 2006; Dawczynski et al., 2007; Neori, 2011) have shifted the focus toward algae which are reservoirs of protein, lipid, carbohydrate, vitamins and pigments and which form the base of the aquatic food chain (Cyrus et al., 2014; Haas et al., 2016; Kiron et al., 2016; Shah et al., 2017; Vadstein et al., 2018). Additionally, although the protein content in the different microalgal species vary, they generally contain all the essential amino acids required for the better growth and survival of the aquatic organisms (Abdulrahman, 2014; Egerton et al., 2018; Vestrum et al., 2018).

In consideration of all these facts, this research study has focused on selecting a protocol for maximum protein extraction from *S. obliquus* (Trup.) Kutz. (SAG 276-3a) biomass after which, the extracted protein has been used to formulate a protein-rich algae meal for the freshwater fishes. After completion of the fish feeding experiments, the algal refinery design shown by Patnaik and Mallick (2015) has been further enriched by addition of the protein component of the test microalga in the valorization process, eventually utilizing the major components present in *S. obliquus* under the optimized condition for production of industrially important products through the designed refinery.

## Materials and Methods

### Microalgal Growth Conditions for Fish Feeding Experiments

Photoautotrophic culture condition of the green microalga, *S. obliquus* (Trup.) Kutz. (SAG 276-3a), was maintained by cultivating the algae in 100 ml of N 11 medium contained in 250 ml Erlenmeyer flasks (Soeder and Bolze, 1981) without aeration. The cultures were maintained under sterile conditions. Light at an intensity of 75 μmol photon m^–2^s^–1^ PAR was continuously supplied for a photoperiod of 14:10 h with pH adjusted at 6.8 and temperature at 25 ± 2°C. Continuous shaking of the culture flasks was done, two to three times a day, to prevent sticking of the culture to the bottom of the flask. To produce enough biomass for the fish feeding experiments, the green microalga, *S. obliquus* was grown in 5 L tubular glass photobioreactors (38 cm height and 15 cm diameter) with working volume of 4 L capacity (courtesy: ICG, Forchungszentrum, Juelich, Germany) as per the protocol detailed by Bagchi and Mallick (2016).

### Selection of Protocol for Protein Extraction From *S. obliquus* Biomass

Five different protocols were used for protein extraction from wet *S. obliquus* biomass.

#### Protocol 1

Extraction of protein was carried out as per the methodology suggested by Barbarino and Lourenco (2005) in which alkali hydrolysis method and ultrapure millipore water were used for extraction of proteins followed by precipitation using trichloroacetic acid. 200 mg of wet microalgal biomass was suspended in 4 mL of ultrapure millipore water and incubated for 12 h at 4°C. After the incubation period, the microalgal suspension was centrifuged at 5000 rpm for 15 min. The supernatant obtained, was collected in another tube and the pellet was re-extracted with 4 mL of 0.1N NaOH containing 0.5% β-mercaptoethanol (v/v), after incubation at room temperature for 1 h with occasional manual shaking. The mixture was then centrifuged again at 5000 rpm for 15 min. The supernatant was collected and mixed with the supernatant obtained in the first phase of the extraction procedure.

The sample was then processed for protein precipitation. Twenty five percent TCA was mixed with the protein containing supernatant in a proportion of (2.5:1, v/v) and kept in an ice bath for 30 min. The mixture was then centrifuged at 5000 rpm for 15 min. The supernatant was discarded and the pellet formed was washed with 10 mL of cold 10% TCA and centrifuged again. The pellet after the second centrifugation was again washed with 5 mL of 5% TCA and centrifuged under the same conditions as mentioned above for separating the protein precipitated at the bottom of the centrifuge tube.

The protein pellet was then processed for quantification using Bradford method (Bradford, 1976). The protein pellets obtained following the above mentioned extraction protocol were suspended in 500 μL of distilled water, and out of this suspension, 100 μL was pipetted into 15 × 1.5 cm test tubes. Five mL of Bradford reagent was added to the test tube and the contents were mixed by vortexing. The absorbance at 595 nm was taken within 1 h in 3 ml cuvettes against a reagent blank prepared from 100 μL of distilled water and 5 mL of Bradford reagent. The concentration of the protein was estimated after comparison with a standard curve.

#### Protocol 2

Protein extraction using Tris-EDTA saturated phenol and precipitation using 0.1 M ammonium acetate prepared in 80% methanol was done as per the protocol suggested by Wang et al. (2006). Microalgal biomass (wet) was homogenized in a mortar and pestle using liquid nitrogen and out of that 200 mg was transferred to a 2 mL tube. The tube was filled with 2 mL of 10% acetone and mixed with the microalgal biomass using a cyclomixture. After 30 min of incubation at room temperature, the mixture was centrifuged at 6000 rpm for 10 min. After centrifugation, the supernatant was discarded and the tube was filled with 2 mL of 0.1 M ammonium acetate prepared in 80% methanol. After proper mixing by vortexing and incubation at room temperature for 15 min, the mixture was centrifuged at 6000 rpm for 10 min. The supernatant was again discarded and the pellet was mixed with 2 mL of 80% acetone. After keeping the mixture undisturbed for 15 min, the acetone was removed after centrifugation under the above mentioned conditions. The pellet was then air dried to remove the residual acetone. To this microalgal pellet, 1 mL of phenol was added, mixed thoroughly and incubated for 5 min at room temperature. The mixture was then centrifuged at 6000 rpm for 5 min and the supernatant containing the proteins dissolved in phenol, was transferred to a fresh 2 mL tube. To this separated supernatant, 1 mL of 0.1 M ammonium acetate prepared in 80% methanol was added, mixed properly and incubated overnight at −20°C for proper protein precipitation. The other day, the mixture was centrifuged at 6000 rpm for 5 min, the supernatant was carefully discarded and the pellet obtained was washed once with 80% methanol and once with 80% acetone. The protein pellet was then processed for quantification using Bradford method (Bradford, 1976).

#### Protocol 3

The protocol recommended by Cilia et al. (2009) was followed for extraction and precipitation of protein from wet *S. obliquus* biomass using 10% TCA prepared in 80% acetone containing 2% β-mercaptoethanol. The wet microalgal biomass (200 mg) was dissolved in 10 mL 10% TCA prepared in 80% acetone containing 2% β-mercaptoethanol. The mixture was mixed thoroughly by vortexing. It was then incubated at −20°C for at least 12 h for protein precipitation. The precipitated protein was separated from the supernatant by centrifugation at 5000 rpm for 15 min and then washed three times in 5 mL of cold acetone. The protein pellet was then processed for quantification using Bradford method (Bradford, 1976).

#### Protocol 4

The protocol suggested by Bhardwaj and Yadav (2013) was followed for extraction of proteins using Tris-HCl saturated phenol and precipitation using 0.1 M ammonium acetate prepared in 80% cold methanol. After homogenization of the wet microalgal biomass using liquid nitrogen in a mortar pestle, 200 mg of homogenized microalgal biomass was suspended in 3 mL of extraction buffer in a 15 mL centrifuge tube, vortexed and incubated for 10 min on ice. Afterward, equal volume, i.e., 3 mL of Tris-HCl saturated phenol was added and the solution was incubated with intermittent shaking at room temperature for 10 min for phase separation to occur. Following this, the solution was centrifuged for 10 min at 5000 rpm. The phenolic phase on the top of the tube, was collected carefully to avoid contact with the interphase, and was transferred to a fresh tube. This phenol phase was then re-extracted with 3 mL of extraction buffer. The sample was shaken and incubated at room temperature for 5 min, after which, it was centrifuged for 10 min at 5000 rpm. The phenol phase, still on the top of the tube, was recovered carefully and transferred to a new tube. To this 10 mL of precipitation solution was added. The contents were mixed by a cyclomixture and then incubated overnight at −20°C. The other day, the protein was finally pelleted after centrifugation at 5000 rpm for 10 min. The pellet was washed once with 80% cold methanol and once with 80% cold acetone. The protein pellet was then processed for quantification using Bradford method (Bradford, 1976).

#### Protocol 5

As proposed by Gerde et al. (2013), protein extraction was carried out by alkali hydrolysis method and ultrapure millipore water followed by precipitation using 2M HCl. 200 mg of wet microalgal biomass was dissolved in 20 mL of 2 M NaOH prepared in ultrapure millipore water containing 0.05% (v/v) of β-mercaptoethanol thus raising the pH of the biomass suspension to pH 11. The mixture was incubated for 5 h at 60°C. The biomass suspension was then centrifuged at 5000 rpm for 15 min and the supernatant collected was acidified using 2M HCl (added drop wise) till the pH was brought down to pH 3.2. After incubation of the acidified mixture at room temperature for 30 min, it was centrifuged at 5000 rpm for 15 min. The supernatant was discarded and the protein pellet was then processed for quantification using Bradford method (Bradford, 1976).

#### Ethics Statement

This study was carried out in accordance with the principles of the Basel Declaration and recommendations of Institutional Animal Ethics Committee (IAEC), IIT Kharagpur. The protocol was approved by the IAEC, IIT Kharagpur.

### Experimental Set-Up for Fish Feeding Experiments

Freshwater fish, namely, rohu (*Labeo rohita*), mrigal (*Cirrhinus mrigala*) and catla (*Catla catla*), were obtained from natural ponds at Agricultural and Food Engineering Department, IIT Kharagpur. Before distributing the fish in different tanks, fish fingerlings (no = 90) were treated with potassium permanganate solution (1 mgL^–1^) for 1 h to remove any external parasites. Then they were randomly divided over six 50 L glass tanks fitted with lights on top of the tanks (15 fish fingerlings of mixed species in each tank). The fingerlings were then allowed to acclimatize to the new growth conditions for 2 weeks. The weight of the fish fingerlings ranged from 3.7 to 4.0 g at the beginning of the acclimatization period. During this period of acclimatization, the fish were fed with their standard diet at 2% of their body weight, only once during the daylight period between 9:00 – 10:30 am. The fecal matter and uneaten feed were removed once every 7 days from each tank by draining approximately 80% of the tank water volume using a siphon pipe and replacing it with an equal volume of clean ground water. The rectangular glass tanks/aquariums were properly aerated with the use of air stones.

#### Experimental Set-Up 1

90 days feeding experiment was conducted with fish fed with three different test diets in three different tanks. The three test diets were standard diet (control), whole *S. obliquus* biomass and standard (control) + whole microalgal biomass (50:50) diets. The control diet consisted of a standard fish meal-based pelleted feed containing fishmeal, groundnut-oil cake, rice bran, wheat flour, and vitamins and minerals mixture in appropriate proportions. Biochemically the control feed had 30% crude protein, 3.5% lipid, and 40% carbohydrate, with the rest of the composition being crude fiber, minerals, ash, and moisture. Contrarily, the whole *S. obliquus* biomass was the harvested microalgal cells without any pre-treatment consisting of 53.2% protein, 12.5% lipid, and 22% carbohydrate. These diets were fed to the fish fingerlings at 2% of their body weight per day. At the start of the experimental set-up 1, the weight of the fish fingerlings ranged from 4.5 to 4.9 g. The amount of feed was calculated and readjusted every 15 days according to change in the body weight. The treatments were executed in duplicate. During this experiment, seven water quality parameters (analyzed as explained below in Section “Analysis of Water Quality Parameters,” daily for pH, temperature and DO, and once every 3 days for the rest of the parameters) and eight growth parameters (analyzed as explained below in Section “Analysis of Fish Growth Performance and Nutrient Utilization Parameters,” once every 15 days) were tested for ensuring proper fish health and management through the progress of the experiment.

#### Experimental Set-Up 2

Another 90 days feeding experiment was conducted with fish fed with three different test diets, standard diet (control), standard (control) + whole microalgal biomass (50:50) diets and standard (control) + extracted microalgal protein (50:50) diets at 2% body weight per day. The treatments were done in duplicate and all the experimental analyses were done in a similar manner as explained above.

#### Experimental Set-Up 3

The third feeding experiment for 90 days was conducted with fish fed with three different test diets, standard diet (control), standard (control) + whole microalgal biomass (50:50) diets and standard (control) + whole microalgal + extracted microalgal protein (25:25:50) diets at 2% body weight per day. The treatments were done in duplicate and all the experimental analyses were done in a similar manner as explained above.

#### Analysis of Water Quality Parameters

Water quality parameters such as temperature, pH, dissolved oxygen (DO) and turbidity were measured using standard instruments. Nitrate, nitrite and total ammonia nitrogen (TAN), contents in the water samples were measured according to the protocols suggested by Nicholas and Nason (1957), Lowe and Evans (1964), and Herbert et al. (1971), respectively.

#### Analysis of Fish Growth Performance and Nutrient Utilization Parameters

##### Body weight gain

The body weight gain (BWG) was calculated as per the formula suggested by Badwy et al. (2008) and was expressed as g fish^–1^. The weight of the fish was measured using a weighing balance (Pioneer Scale Industries, Kolkata, India). Fish were caught using a fish net and were then placed on a weighing balance. Weight of the fish was measured after the fish became stable on the balance. An average value of the wet weight was taken after noting down three independent readings. The fish fingerlings were not anesthetized during their weight measurement.

Body weight gain (g fish^–1^) = Weight (g) at the end of the experimental period – weight (g) at the beginning of the experimental period.

##### Specific growth rate

The specific growth rate (SGR) of the fish was calculated as per the formula suggested by Badwy et al. (2008).

Specific growth rate (SGR)=(ln W-1ln W)0/(t-2t)1.

where W_1_ was the final body weight of the fish after time t_2_ and, W_0_ was the initial body weight of the fish at time t_1_.

##### Feed conversion ratio (FCR)

The FCR was calculated as per the formula suggested by Siddhuraju and Becker (2003). Total feed fed to the fishes for every 15 days period was calculated and expressed as (g fish^–1^). Total weight gained by the fishes (in each tank) after every 15 days of feeding was measured as per the method detailed for measurement of BWG.

Feed conversion ratio (FCR)=Total feed fed (g fish)-1/Total wet weight gain (g).

##### Protein efficiency ratio (PER)

The PER was calculated as per the method suggested by Siddhuraju and Becker (2003). Fish were caught in a fish net and euthanized by rapid chilling. Fish flesh were then collected and grounded using liquid nitrogen. Protein extraction using ultrapure water, NaOH and TCA was done following Barbarino and Lourenco (2005). Protein estimation was done following Bradford (1976).

=Wet weight gain (g fish)-1/Amount of protein fed (g fish)-1.

##### Apparent net lipid utilization (ANLU)

The ANLU was calculated as per the formula suggested by Becker et al. (1999). Fish were first caught in a fish net and euthanized by rapid chilling. Fish flesh was then collected and grounded using liquid nitrogen. Lipid extraction using methanol and chloroform was done following Bligh and Dyer (1959). The amount of lipid obtained/lipid yield was calculated by subtracting W_1_ (weight of the empty vial) from W_2_ (weight of the vial containing the extracted lipid) and was expressed as gL^–1^.

ANLU=[final fish body lipid (g)-initial fish body lipid (g)/crude lipid fed (g)]×100.

##### Protein productive value (PPV)

The PPV was calculated using the formula suggested by Siddhuraju and Becker (2003).

Protein productive value (PPV)

=[gain in fish body protein (g)/crude protein fed (g)]×100.

##### Metabolic growth rate (MGR)

The MGR was calculated as per the formula suggested by Becker et al. (1999).

Metabolic growth rate (MGR)

=live body weight gain (g)/[(initial body weight (g)/1000)80.+(final body weight (g)/1000)/0.82]day-1.

##### Omega-3 Fatty acid analysis

Fish were first caught in a fish net and euthanised by rapid chilling. Fish flesh was collected and grounded using liquid nitrogen. Lipid extraction was done following Bligh and Dyer (1959) following which the amount of lipid obtained was expressed as gL^–1^. Transesterification of the extracted lipids was done following Mandal et al. (2013) and analyzed using GC-MS as per the method detailed in Patnaik and Mallick (2015).

### Proposition for an Algal Refinery

This manuscript being a sequel to the previously published paper by Patnaik and Mallick (2015), the test microalga for this part of the study has been grown under the optimized condition for designing the algal refinery. The optimized condition comprised of *S. obliquus* grown in N 11 medium supplemented with 0.17% acetate, 0.17% citrate and 0.4 g L^–1^ nitrate and incubated for a period of 9 days in a temperature controlled culture room. The sequential extraction of all the components from the test microalga other than protein was done as per the methods detailed in Patnaik and Mallick (2015) whereas the protein part was extracted following the method standardized in this manuscript.

To find out the correct sequence of extraction of different components (so as to avoid any negative impact on the extraction yields of different components) four different sequences were tried as shown in Table 1.

**TABLE 1:** Variation in the yields of the different microalgal components when arranged in different sequential positions.

**Sequence 1**	**Sequence 2**	**Sequence 3**	**Sequence 4**
β-carotene yield (0.061 ± 0.07 g)	β-carotene yield (0.054 ± 0.03 g)	β-carotene yield (0.063 ± 0.09 g)	β-carotene yield (0.057 ± 0.05 g)
↓	↓	↓	↓
Lipid yield (54.78 ± 1.97 g)	Carbohydrate yield (28.77 ± 1.32 g)	Lipid yield (56.16 ± 1.85 g)	Protein yield (9.79 ± 0.72 g)
↓	↓	↓	↓
Carbohydrate yield (30.05 ± 1.63 g)	Lipid yield (32.21 ± 1.95 g)	Protein yield (6.43 ± 0.46 g)	Lipid yield (55.83 ± 1.94 g)
↓	↓	↓	↓
Protein yield (5.41 ± 0.53 g)	Protein yield (2.42 ± 0.22 g)	Carbohydrate yield (26.71 ± 0.78 g)	Carbohydrate yield (29.78 ± 1.28 g)

*Values represent the mean ± SE of data based on three independent determinations.*

### Statistical Analysis

Microsoft Excel (Microsoft Corporation, United States) was used for graphical representations and determination of the standard error values (obtained from experiments carried out by using three independent cultures to confirm their reproducibility) for all experiments related to standardization of protein extraction protocols and microalgal refinery design. Fish feeding experiments were done in duplicate. Statistical analysis and graphical representations of the fish growth and nutrient utilization studies were also done using Microsoft Excel (Microsoft Corporation, United States).

## Results and Discussion

### Selection of Protocol for Protein Extraction From *S. obliquus* Biomass

For extraction of protein from *S. obliquus* biomass, five different protocols were tested by following the methods described in Section “Selection of Protocol for Protein Extraction From *S. obliquus* Biomass.” The extracted proteins were then analyzed using Bradford method (Bradford, 1976). The protein yield from the whole *S. obliquus* biomass grown under control condition for a period of 21 days was found to be 0.65 gL^–1^ (53.2% dcw), as measured before the initiation of the extracting protocols. Of all the protocols tested, Protocol 1 proved to be the most efficient. The selected protocol produced maximum extraction of protein up to 0.58 gL^–1^ (47.5% dcw) after 21 days of incubation. So, this protocol was able to extract 89% of the protein present in the whole *S. obliquus* biomass under control condition. As compared to the other protocols, Protocol 1 was found to extract maximum amount of proteins, probably due to the presence of more water-soluble proteins in the test microalgal biomass (Algae PARC, 2016), the denaturation of the disulfide bonds in the protein molecules by β-mercaptoethanol (Nelson and Cox, 2000), and the disruption of the solvation layers of the proteins and their partial denaturation, thus exposing even more hydrophobic surface to the solvent and enhancing the hydrophobic aggregation of the protein molecules by using TCA (trichloroacetic acid) (Barbarino and Lourenco, 2005; Cilia et al., 2009).

**FIGURE 1:**
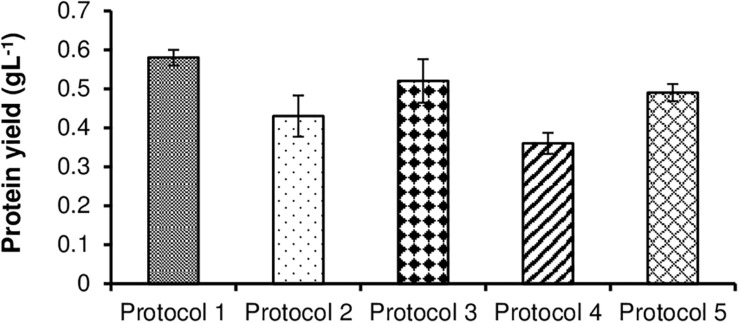
Comparative representation of the amount of protein extracted from *S. obliquus* biomass using different extraction protocols.

Hence, this experiment suggested the significance of a thorough knowledge of the type of proteins present inside the test organism along with their solubility and structure for selecting/designing an efficient protein extraction protocol. Additionally, the selected method should be compliable for a broad range of microalgal species, in both, their wet and lyophilized forms. Furthermore, the extraction protocol should not be extremely harsh so as to jeopardize the complete protein functionality ultimately compromising with quantification of the extracted protein using dye-based reactions. Another important point worth keeping in mind is that, the extraction protocol alone, ultimately decides the use to which the extracted protein can be put to in a refinery design. The results of different protein extraction protocols used have been given in Figure 1.

### Fish Feeding Experiment

#### Trial Experiment

After selection of the protein extraction protocol, experiments to formulate a protein-rich algae meal for substitution in appropriate quantities in the standard fish diet, was carried out. Trial experiment for investigating the ability of the freshwater fish species, rohu (*Labeo rohita*), mrigal (*Cirrhinus mrigala*), and catla (*Catla catla*), to feed on *S. obliquus* whole biomass as their only feed resource was performed for a period of 30 days. Additionally, the ability of the dried microalgal biomass pellet to sink to the bottom and remain undispersed till eaten by the fishes, was also checked. It was observed that the microalgal biomass was being consumed by the fishes with significant improvement in growth without much wastage of the supplied feed. The water quality parameters measured, were found to be well maintained within the tolerable limits of the fishes during the trial period.

#### Experimental Set-Up 1

After confirmation of the acceptability of *S. obliquus* biomass as a feed supplement by the freshwater fishes, the first experimental set-up for 90 days was conducted with fish fed with three different test diets at 2% body weight per day. The body weight of the fish in the test tanks on the 0 day (on the day of the start of the experiment) was 4.5 ± 0.72, 4.9 ± 0.65, and 4.7 ± 0.69 g in the tank fed with the standard diet (control), whole *S. obliquus* biomass diet, and standard + whole microalgal biomass (50:50) diet, respectively. The control diet consisted of a standard fish meal-based pelleted feed with 30% crude protein, 3.5% lipid, and 40% carbohydrate, with the rest of the composition being crude fiber, minerals, ash, and moisture. The *S. obliquus* biomass consisted of 53.2% protein, 12.5% lipid, and 22% carbohydrate.

##### Fish growth performance and nutrient utilization parameters

**FIGURE 2:**
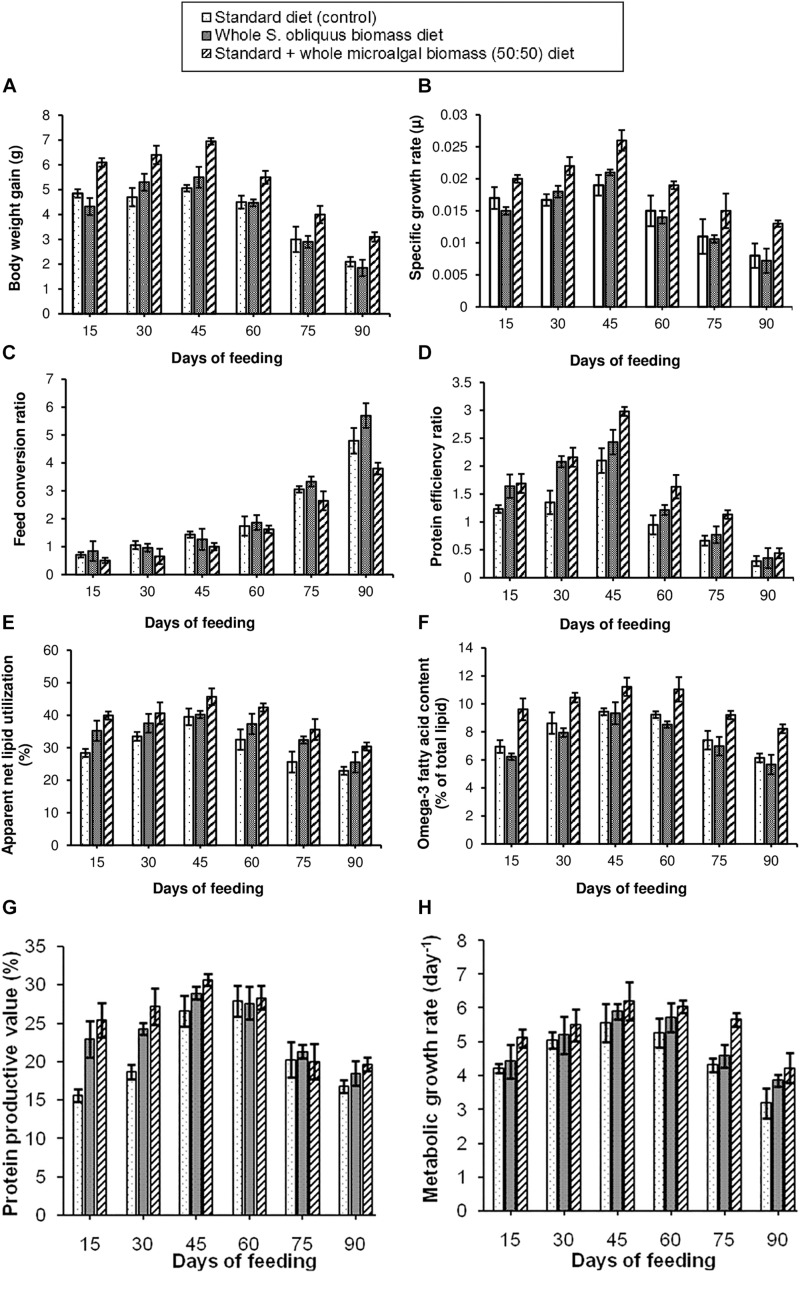
Comparative representation of the **(A)** body weight gain, **(B)** specific growth rate, **(C)** feed conversion ratio, **(D)** protein efficiency ratio, **(E)** apparent net lipid utilization, **(F)** omega-3 fatty acid content, **(G)** protein productive value, and **(H)** metabolic growth rate in three different diet conditions.

The growth parameters for assessing the effect of different diets on the freshwater fishes was studied as per the methods described in Section “Analysis of Fish Growth Performance and Nutrient Utilization Parameters.” Eight different growth performance and nutrient utilization parameters i.e., BWG, SGR, FCR, PER, ANLU, omega-3 fatty acid content, PPV and MGR were observed. The results of the growth study have been shown in Figures 2A–H. It was observed that, all the above parameters showed maximum improvement in the fishes fed with 50:50 ratio of standard + whole *S. obliquus* biomass diet. Under this diet condition, maximum BWG of 6.95 g was observed in the third fortnight, i.e., within 30–45 days of feeding. The maximum SGR when fed with this diet was 0.026 μ. Out of the three test diets, this diet was observed to have the lowest FCR value, indicating the cost-effectiveness of the formulated diet. Hence it was implied that the maximum portion of 50:50 ratio of standard + whole *S. obliquus* biomass diet was able to be assimilated in the body of the fishes. Similarly, the PER value of this diet was found to be the highest among all three diet conditions indicating higher contribution of the protein in the standard + whole *S. obliquus* biomass feed toward raising the weight of the fish. It was also observed that the proportion of the lipid assimilated into the body of the fish out of the total crude lipid fed, i.e., ANLU was maximum (45.7%) in the third fortnight of feeding the 50:50 ratio of standard + whole *S. obliquus* biomass diet. Hence the omega-3 fatty acid content of 11.2% was also found to be maximum during that period. Similar to these observations, the PPV, i.e., the amount of protein gained by the fish after being fed with the test diet was found to be maximum (30.6%) within 30–45 days. With all the growth performance and nutrient utilization parameters showing maximum value in the third fortnight of feeding with 50:50 ratio of standard + whole *S. obliquus* biomass diet, the MGR was consequently found to be maximum during the same time period. This finding was found to be well in agreement with Radhakrishnan et al. (2015) in which 50% inclusion of *C. vulgaris* in the control diet, increased the growth parameters in *Macrobrachium rosenbergii.* Higher feeding rate and better assimilation of the protein fraction of the microalgal diet in the prawn were reasons cited by the authors for such an observation. The other two diets, i.e., standard (control) diet and whole *S. obliquus* biomass diet showed comparatively poorer performance as assessed through the above mentioned parameters. The 100% inclusion level of whole *S. obliquus* biomass diet in this research study must have reduced the palatability of the feed thus showing comparatively lower growth (Figure 2). Contrary to this observation, 100% inclusion percentage of *Spirulina platensis* was found to improve the specific growth rate and PER of the freshwater fish, *Labeo rohita* as observed in the report of Nandeesha et al. (2001). Algal inclusion percentages of <50% have also been recommended by few researchers (Teimouri et al., 2013; Ghosh and Mitra, 2015; Basri et al., 2015) for improving growth and/or functional component accumulation in some fish species. The examples cited, thus justify the species-specific dependence of both, the microalga (to be included in the fish diet) and the fishes (to be fed with the microalga included diet) on each other for improvement in the fish growth and nutrient utilization parameters. Moreover, standardization of the inclusion percentage of protein-rich algal meal in the diets of the freshwater fishes is also suggested to be of utmost importance.

##### Water quality parameters

The water quality parameters of the fish tanks were analyzed as per the protocols described in Section “Analysis of Water Quality Parameters.” As the experiment was carried out from September to November, the temperature gradually saw a declining trend due to gradual decline in the atmospheric temperature in this geographical locale. The three parameters, i.e., temperature, pH and DO were not found to have any significant difference in the fish tanks administered with the three test diets. However, in case of turbidity, TAN, nitrate and nitrite content, the fish tanks provided with standard diet, showed higher values of the said parameters (data not shown) which could have been due to the accumulation of the residual feed and inadequate oxidation of the fecal matter (Ghosh and Mitra, 2015).

#### Experimental Set-Up 2

As 50% inclusion of whole *S. obliquus* biomass showed the best results in the first set-up (Figure 2), the second set-up was designed to reconfirm the results of the first set-up, and in addition explore the effect of 50% inclusion of extracted microalgal protein in the standard diet. Another 90 days feeding experiment was conducted with fish fed with three different test diets, standard diet (control), standard + whole microalgal biomass (50:50) diet, and standard + extracted microalgal protein (50:50) diet at 2% body weight per day. The body weight for the fish in the test tanks on the 0 day (on the day of the start of the experiment) was 4.6 ± 0.83, 5.3 ± 0.79, and 5.8 ± 0.72 g, respectively. The experimental set-up was similar to that of the first experiment.

##### Fish growth performance and nutrient utilization parameters

**FIGURE 3:**
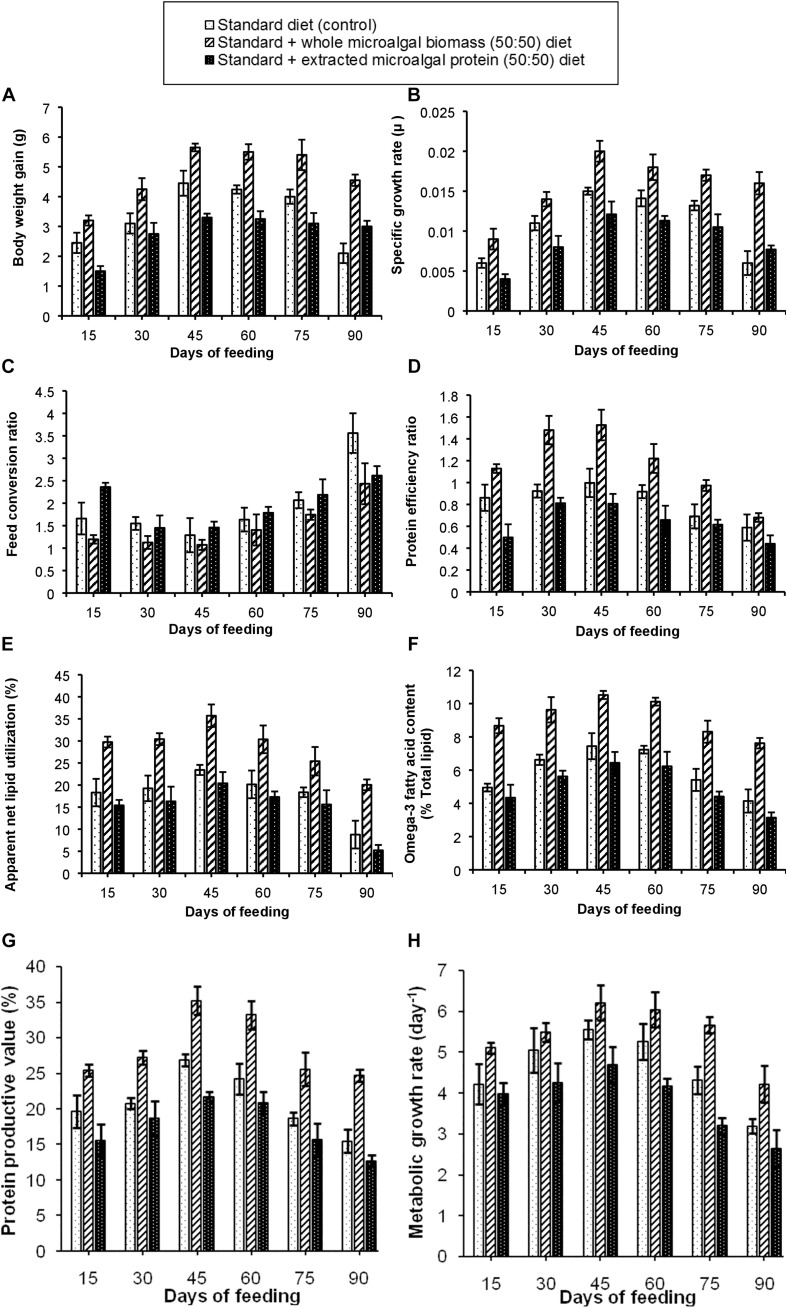
Comparative representation of the **(A)** body weight gain, **(B)** specific growth rate, **(C)** feed conversion ratio, **(D)** protein efficiency ratio, **(E)** apparent net lipid utilization, **(F)** omega-3 fatty acid content, **(G)** protein productive value, and **(H)** metabolic growth rate in three different diet conditions.

The results of the experiment have been shown in Figures 3A–H. Out of the three test diets, the standard + whole *S. obliquus* biomass (50:50) diet in this experimental set-up also, showed the best growth performance. It was observed that, BWG of the fishes in these test tanks increased gradually with maximum BWG of 5.65 g in the third fortnight of feeding. The SGR of the fish fed on 50:50 ratio of control and whole *S. obliquus* biomass diet was also found to be maximum (0.02 μ), with a gradual decline in the subsequent time periods. An observation of the FCR, PER and PPV values further attested the best growth performance in the fish fed with this test diet. The percentage of ANLU and omega-3 fatty acid content, were also found to be maximum between 30 to 45 days of feeding with their respective values being, 35.7 and 10.5%. The diet formulation with standard + extracted microalgal protein (50:50) diet was however, not found to be very significant as compared to the other two test diets which could be due to the lower palatability of this diet which was clearly demonstrated in the poorer growth performance in the fishes administered with this diet, as compared to the control and 50% whole *S. obliquus* biomass included diet. Such an experiment with inclusion of only extracted plant or animal protein has not been carried out by any researcher, to the extent of our knowledge and hence is not available for reference.

##### Water quality parameters

The temperature, as recorded daily was found to drift toward higher degrees gradually due to the change in season during the months of December–February, in this geographical locale. The three parameters, i.e., temperature, pH and DO were not found to have any significant difference in the fish tanks administered with the three test diets. However, in case of turbidity, TAN, nitrate and nitrite content, the fish tanks provided with standard + extracted microalgal protein (50:50) diet, showed a higher value of the said parameters due to higher accumulation of the uneaten feed. Comparatively, the fish tanks with the standard diet showed lower wastage of the administered feed, hence lower values of turbidity, TAN, nitrite and nitrate but the lowest values of the three measured parameters was observed in the tanks with the standard + whole *S. obliquus* biomass (50:50) diet. These four parameters were found to increase gradually in the test tanks with increased days of feeding (data not shown).

#### Experimental Set-Up 3

As 50% inclusion of the extracted microalgal protein along with 50% standard diet, did not show any improvement in the growth parameters of the freshwater fish varieties in the second experimental set-up, the proportion of the standard diet was reduced to 25 and 25% of whole *S. obliquus* biomass was included along with 50% extracted algal protein in the third set-up, to increase the palatability of the test diet for consumption by the fishes. The third experimental set-up for 90 days was carried out in a similar way as was done in the other two experimental set-ups. The weight of the fishes before the start of the experiment were 5.6 ± 0.72, 5.9 ± 0.66, and 5.5 ± 0.71 g in the fish tanks administered with standard diet (control), standard + whole microalgal biomass (50:50) diet, and standard + whole microalgal biomass + extracted microalgal protein diet (25:25:50) diet, respectively.

##### Fish growth performance and nutrient utilization parameters

**FIGURE 4:**
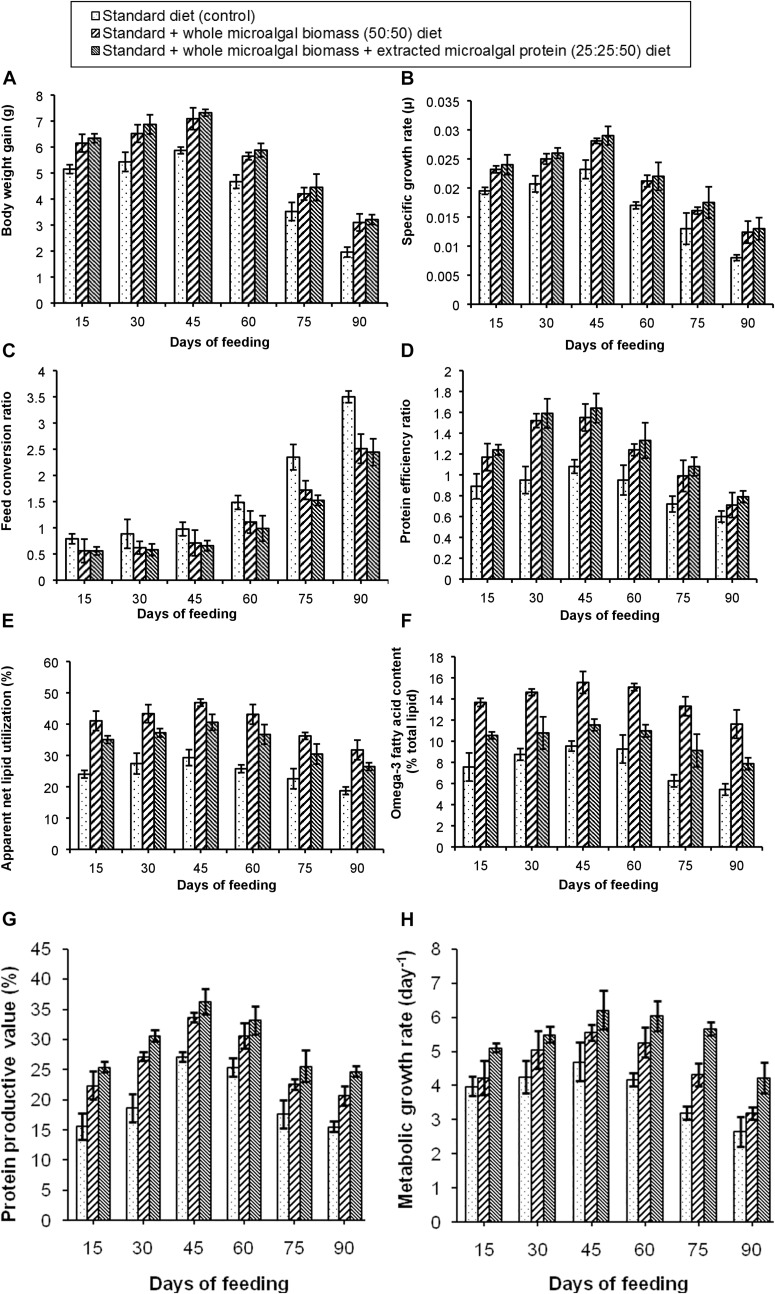
Comparative representation of the **(A)** body weight gain, **(B)** specific growth rate, **(C)** feed conversion ratio, **(D)** protein efficiency ratio, **(E)** apparent net lipid utilization, **(F)** omega-3 fatty acid content, **(G)** protein productive value, and **(H)** metabolic growth rate in three different diet conditions.

The results of the experiment have been shown in Figures 4A–H. Out of the three test diets, the standard + whole microalgal biomass + extracted microalgal protein (25:25:50) diet showed the best growth performance. It was observed that, BWG of the fishes in this test tank increased gradually with maximum BWG (7.3 g) in the third fortnight of feeding. The SGR of the fish fed on the same diet was also found to be 0.028 μ. Higher incorporation of protein due to higher nutrient digestibility (Nandeesha et al., 1994; Mustafa and Nakagawa, 1995) could have led to this increased body weight gain and SGR in the selected test diet. Although the microalgal protein during extraction and precipitation could have been denatured, the amino acids to which it was broken down to were successfully incorporated in the body of the fishes (Sward, 2014). While this diet condition showed maximum gain in body weight, the BWG in case of standard + whole microalgal biomass (50:50) diet was found to be only marginally different. An observation of the FCR and PER values further confirmed the best growth performance in the fish fed with this test diet. The percentage of ANLU and omega-3 fatty acid content, were however, found to be maximum within 30–45 days of feeding with the standard + whole microalgal biomass (50:50) diet, their respective values being, 46.8 and 15.5%. But in case of PPV and MGR, maximum improvement was seen in the third fortnight of feeding the fishes with standard + whole microalgal biomass + extracted microalgal protein (25:25:50) diet. The maximum PPV was recorded to be 36.3% and maximum MGR was found to be 6.21 day^–1^, thus, attesting the use of the microalgal protein as an aquafeed, during the formation of an algal refinery.

##### Water quality parameters

The water quality parameters in the fish tanks were analyzed as per the protocols described in Section “Analysis of Water Quality Parameters.” Temperature, as recorded daily was found to drift toward higher degrees gradually due to the change in change in atmospheric temperature during March – May. As observed in the other two experimental set-ups, these three parameters, i.e., temperature, pH and DO, were not found to have any significant difference in the fish tanks administered with the three different test diets. However, in case of turbidity, TAN, nitrate and nitrite content, the fish tanks provided with standard diet, again showed higher values of the said parameters. Comparatively, the fish tanks with the standard + whole microalgal + extracted microalgal protein (25:25:50) and standard + whole microalgal biomass (50:50) diets showed lower wastage of the administered feed, hence lower values of turbidity, TAN, nitrite and nitrate, varying marginally from each other (data not shown).

Fish feeds account for a significant portion of the costs in an aquaculture enterprise. Hence, to minimize the feed costs while simultaneously looking into environmental sustainability, accurate information about the nutritional requirement of the fishes is imperative so as to adopt balanced diet formulations and feeding practices for their optimal growth and development. Fishes mostly require diets composed of proteins, lipids and carbohydrates for good growth and maintenance, however, amongst them, proteins are required in maximum proportions, comprising about 65–70% of the fish muscle. Proteins consumed by the fishes not just provide the essential and non-essential amino acids for their muscle formation and enzymatic function, but also provide energy for their maintenance. Insufficient amounts of protein in the fish diets lead to a decline or diminution of their growth parameters probably due to restricted supply of proteins to more vital organs and tissues. While, excessive amounts of protein in the fish diets result in accelerated energy requirements, higher costs, more nitrogenous waste excretions and decreased fish growth. Moreover, as proteins are the most expensive item in the commercially available fish feeds, it is indeed wise to include optimum dietary protein levels in their diets, thus avoiding biological and economical loss without compromising with their growth and maintenance. Furthermore, as fish growth also depends on the effectiveness of the withholding time of the dietary protein, optimum protein proportions for each species including their cultivation phase needs should also be determined for maximum profitability.

### Proposition of an Integrated Sequential Processing Approach for an Algal Refinery

#### Sequential Production of Biodiesel and Various Co-products

##### Selection of the sequence for maximum extraction of the microalgal components

The extraction of the components from *S. obliquus* biomass in this part of the study was done under the optimized culture condition as detailed in Section “Proposition for an Algal Refinery.” During a sequential extraction process, the biggest problem posed is by the interference of the solvents and the methodologies used for extraction of one component on the yield of the other microalgal components. Hence proper placement of the lipids and other components one after the other, without much effect on their extracted yields is essential. For this purpose four different sequences were tried. A display of the variation in the yields of the different components when arranged in different sequential positions has been given in Table 1.

Before elaborating on the data given in Table 1, a mention of the yields of the different components (each component extracted individually from the whole *S. obliquus* biomass without any sequential processing) obtained under the optimized condition from 100 g of dry *S. obliquus* biomass is important. The β-carotene, lipid, carbohydrate and protein yield, extracted individually was 0.06, 56.2, 30.2, and 10.2 g, respectively.

From the Table above, it was observed that when sequence 1 was followed, the yields of β-carotene, lipid and carbohydrate were insignificantly affected, but the protein yield was reduced drastically to 5.4 g from 1 Kg wet (≈100 g dry) *S. obliquus* biomass. This observation indicated that neither did the use of acetone for β-carotene extraction affect the lipid yield from the microalgal biomass as the amount of lipid obtained after β-carotene extraction was nearly the same as that obtained directly from the test microalga, nor did the solvents acetone, chloroform and methanol affect the carbohydrate yield, extracted sequentially after β-carotene and lipid, but the positioning of protein extraction at the end of the sequential process affected the protein yield severely. The reduced yield of protein could possibly have been due to leakage during acid hydrolysis of the biomass for carbohydrate extraction. The loss in biomass during the sequential extraction procedure was however, found to be negligible. In case of sequence 2, a reduction in both, the lipid and protein yield to 32.2 and 2.4 g, respectively was observed when the lipid and protein extractions were preceded by carbohydrate extraction from the dry microalgal biomass due to loss of lipids and proteins during acid hydrolysis of the dry microalgal biomass. β-carotene extraction on the top of the sequence was not found to be responsible for this observation which can also be confirmed from the results of the first sequential extraction process.

The results of the third sequential extraction process showed that if β-carotene and lipid extraction are followed by protein extraction instead of carbohydrate extraction then the yield of the extracted carbohydrate was reduced marginally to 26.7 g but the yield of the protein obtained was still significantly lower than that obtained directly from the whole *S. obliquus* biomass under the optimized condition which was due to the interference of the solvents used during lipid extraction in the extraction of proteins using ultra-pure water (Wessel and Flugge, 1984), although after being placed in the third position of the sequential extraction process the protein yield showed an increase to 6.4 g.

However, implementation of the fourth sequential extraction process resulted in maximum yields of the microalgal components which were unaffected by any form of loss due to use of solvents or methods of cell hydrolysis. The chosen sequence extracted β-carotene followed by protein, lipid and carbohydrate. β-carotene and protein extraction were done from wet *S. obliquus* biomass, but the data presented, has been given in terms of dry cell weight for a better representation of the microalgal biomass composition.

##### Microalgal refinery

**FIGURE 5:**
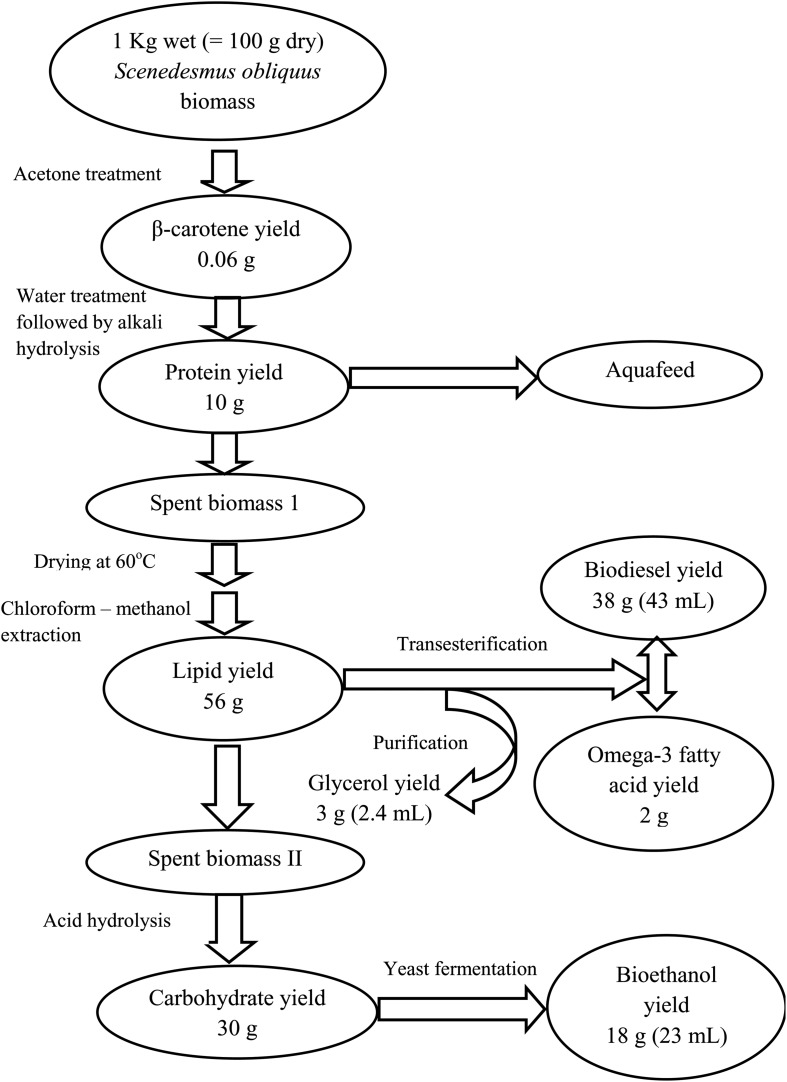
Modified schematic representation of the detailed process for sequential production of biodiesel and other industrially valuable co-products from *S. obliquus* biomass.

The design of the proposed algal refinery has been shown in Figure 5. The harvested wet biomass of *S. obliquus* was treated with acetone for β-carotene extraction followed by protein extraction using ultrapure water and alkali hydrolysis of the microalgal cells. The *S. obliquus* biomass was then dried at 60°C followed by lipid extraction using a binary solvent system (chloroform-methanol). The de-fatted biomass was then subjected to acid hydrolysis for the breakdown of the complex polysaccharides to simple monomers for bioethanol production through fermentation by *Saccharomyces cerevisae*. 1 Kg wet (≈100 g dry) *S. obliquus* biomass under the optimized condition yielded 0.06 g of β-carotene, 10 g of protein, 38 g (43 mL) of biodiesel, 2 g of omega-3 fatty acid, 3 g (2.4 mL) of glycerol and 18 g (23 mL) of bioethanol. As shown in Figure 5, during conversion of carbohydrates to ethanol, it was observed that ∼60% of the total carbohydrate contained in the microalgal biomass under the optimized condition was converted to bioethanol. Although theoretically, 50% conversion of the carbohydrates in the microalgal cells is possible, conversions of carbohydrates to bioethanol by >50% may reflect fermentation of additional carbohydrates beside glucose and mannose, which was not accounted for in the theoretical calculations (Laurens et al., 2015). Hence 70% of the test microalgal biomass was successfully used for production of biodiesel and other value-added products by using an algal refinery approach.

The approach of algal refinery as a method to reduce the cost of the algal biodiesel by co-production of various value-added chemicals, antioxidants, fertilizers etc. is the most up-coming strategy suggested for the economic viability of the microalgal biodiesel. This concept is a relatively new and novel idea where abundant literature is not available although fragmented data on some feedstocks are present. A report by Dong et al. (2016) on the valorization of *Scenedesmus acutus* by simultaneous production of bioethanol and biodiesel in a combined algal processing method has shown to utilize nearly 66% of the microalgal biomass for energy generation thus bringing down the cost of the total energy produced by 9% from $10.86/GGE (Gallons of Gasoline equivalent) to $9.91/GGE. In another report by Marinho et al. (2016), the macroalga *Saccharina latissima* in a biorefinery approach has been shown to produce succinic acid after which the leftover solid residue has been tested for their total phenolic compounds and macronutrients (Ca, K, Na, Mg, P, N, and Fe) content. These phenolic compounds and macronutrients contained in the leftover residue have been proposed to be used as antioxidants and fertilizers, respectively, thus, demonstrating the potential of ∼70% of the *S. latissima* biomass to be converted to value-added products. In the present study, a successful conversion of 70% of the *Scenedesmus obliquus* biomass into industrially important products such as β-carotene, protein for aqua-feed, biodiesel, omega-3 fatty acid, glycerol and bioethanol through defined sequential processing steps has been demonstrated unlike the report by Marinho et al. (2016) in which conversion of the phenolic compounds and macronutrients to antioxidants and fertilizers has not been carried out. Additionally, the production of multiple high-value products as shown in the refinery design further confirms the comparative richness and relevance of the present research study in providing an economically sustainable model in future during application of the refinery design in a pilot scale.

Cost-intensive production process of microalgal biodiesel is a major obstacle in its commercialization. Hence efforts should be made to reduce the costs associated with microalgal production and conversion of the algal intermediates to biodiesel. But merely improving the biomass productivity or augmenting the quantity of lipids in microalgae is not expected to bring enough cost reductions. For further progress, complete utilization of all algal components is essential. Previous research reports have shown substantial reductions in microalgal biofuel production costs by applying combined algal processing techniques through simultaneous production of proteins, carbohydrates and other valuable cellular compounds. But, the production of higher value co-products differs depending on the algal biomass composition. Therefore for making maximum profit from algal biodiesel production, decisions should be made judiciously regarding the algal strains possessing maximum potential for producing different high-value products; methodologies used for sequential extraction of different cellular components so as to incur minimal losses in terms of loss of compounds; the potential for nutrients and solvents recycling; and degradation or denaturation of the cellular compounds during the extraction process. Furthermore, colocation of the refinery units with algal farms can reduce the transportation and pre-processing costs. Algal refineries are expected to lead to high quality job creation and energy independence by bridging the gap between high-value small-market products and low-value large-market products. Hence extensive research on these refinery designs is expected to be of immense benefit.

## Conclusion

An algal refinery is a concept that is being followed nowadays as a strategy to reduce the high costs incurred during microalgal biodiesel production. Hence, a study with a sequential processing design for production of biodiesel and other industrially important products such as β-carotene, protein, biodiesel, omega-3 fatty acid, glycerol and bioethanol, might appear conceptually incomplete without an assessment of the economics of the entire process to ascertain its viability and sustainability. But the experiments having been carried out in the laboratory under controlled culture condition, calculation of the costs incurred in the entire process will not be realistic. Therefore, scaling up of the present study to pilot scale under outdoor condition should be carried out in future to valuate and validate the economic sustainability of the designed algal refinery. Furthermore, as the present study demonstrates conversion of 70% of the *S. obliquus* biomass into industrially important products, efforts to convert the remaining 30% of the microalgal biomass into value-added products through improved methodologies to valorize the whole algal biomass with minimal loss would further enrich the proposed strategy in future.

## Data Availability

All datasets generated for this study are included in the manuscript/the supplementary files.

## Ethics Statement

The animal study was reviewed and approved by Institutional Animal Ethics Committee, IIT Kharagpur.

## Author Contributions

RP wrote the article, conducted all the three fish feeding experiments including the experiments for the final algal refinery design, analyzed and interpreted the results of all the experiments. NS performed the first two set-ups of the experiments during which he analyzed four growth parameters and seven water quality parameters. SB also contributed to this work through his help during data analysis. NM and PR supervised the design of the experiments. NM checked the manuscript. All authors have read and approved the final manuscript.

## Conflict of Interest Statement

The authors declare that the research was conducted in the absence of any commercial or financial relationships that could be construed as a potential conflict of interest.

## References

[B1] Abdulrahman N. M. (2014). Evaluation of *Spirulina* spp. as food supplement and its effect on growth performance of common carp fingerlings. *Int. J. Fish. Aquat. Stud.* 2 89–92.

[B2] Algae PARC, (2016). *Development of Protein Isolation Methods from Microalgae.* Available at: https://www.wur.nl/en/show/Development-of-protein-isolation-methods-from-microalgae.htm (accessed January 10, 2017).

[B3] Badwy T. M. Ibrahim E. M. Zeinhom M. M. (2008). “Partial replacement of fish meal with dried microalga (*Chlorella* spp and *Scenedesmus* spp) in Nile Tilapia (*Oreochromis niloticus*) diets,” in *Proceedings of the 8th International Symposium on Tilapia in Aquaculture*, (Egypt: Central Laboratory for Aquaculture Research), 801–811.

[B4] Bagchi S. K. Mallick N. (2016). Carbon dioxide biofixation and lipid accumulation potential of an indigenous microalga *Scenedesmus obliquus* (Turpin) Kützing GA 45 for biodiesel production. *RSC Adv.* 6 29889–29898. 10.1039/c6ra02811j

[B5] Banerjee C. Dubey K. K. Shukla P. (2016). Metabolic engineering of microalgal based biofuel production: prospects and challenges. *Front. Microbiol.* 7:432. 10.3389/fmicb.2016.00432 27065986 PMC4815533

[B6] Barbarino E. Lourenco S. O. (2005). An evaluation of methods for extraction and quantification of protein from marine macro- and microalgae. *J. Appl. Phycol.* 17 447–460. 10.1007/s10811-005-1641-4

[B7] Basri N. A. Muhamad S. S. R. Matanjun P. Mohammad Noor N. Shapawi R. (2015). The potential of microalgae meal as an ingredient in the diets of early juvenile Pacific white shrimp, *Litopenaeus vannamei*. *J. Appl. Phycol.* 27 857–863. 10.1007/s10811-014-0383-6

[B8] Becker K. Schreiber S. Angoni C. Blum R. (1999). Growth performance and feed utilisation response of *Oreochromis niloticus* × *Oreochromis aureus* hybrids to L-carnitine measured over a full fattening cycle under commercial conditions. *Aquaculture* 174 313–322. 10.1016/s0044-8486(99)00019-8

[B9] Bhardwaj J. Yadav S. K. (2013). A common protein extraction protocol for proteomic analysis: horse gram a case study. *Am. J Agric. Biol. Sci.* 8 293–301. 10.3844/ajabssp.2013.293.301

[B10] Bligh E. G. Dyer W. J. (1959). A rapid method of total lipid extraction and purification. *Can. J. Biochem. Physiol.* 37 911–917. 13671378 10.1139/o59-099

[B11] Borowitzka M. A. (2013). High-value products from microalgae—their development and commercialisation. *J. Appl. Phycol.* 25 743–756. 10.1007/s10811-013-9983-9

[B12] Bradford M. M. (1976). A rapid and sensitive method for the quantitation of microgram quantities of protein utilizing the principle of protein-dye binding. *Anal. Biochem.* 72 248–254. 10.1006/abio.1976.9999 942051

[B13] Camacho-Rodríguez J. Macías-Sánchez M. D. Cerón-García M. C. Alarcón F. J. Molina-Grima E. (2017). Microalgae as a potential ingredient for partial fish meal replacement in aquafeeds: nutrient stability under different storage conditions. *J. Appl. Phycol.* 30 1049–1059. 10.1007/s10811-017-1281-5

[B14] Cilia M. Fish T. Yang X. Mclaughlin M. Thannhauser T. W. Gray S. (2009). A comparison of protein extraction methods suitable for gel-based proteomic studies of aphid proteins. *J. Biomol. Tech.* 20 201–215. 19721822 PMC2729484

[B15] Cyrus M. D. Bolton J. J. Scholtz R. Macey B. M. (2014). The advantages of *Ulva* (Chlorophyta) as an additive in sea urchin formulated feeds: effects on palatability, consumption and digestibility. *Aquac. Nutr.* 21 578–591. 10.1111/anu.12182

[B16] Dawczynski C. Schubert R. Jahreis G. (2007). Amino acids, fatty acids, and dietary fibre in edible seaweed products. *Food Chem.* 103 891–899. 10.1016/j.foodchem.2006.09.041

[B17] Delrue F. Setier P. A. Sahut C. Cournac L. Roubaud A. Peltier G. (2012). An economic, sustainability, and energetic model of biodiesel production from microalgae. *Bioresour. Technol.* 111 191–200. 10.1016/j.biortech.2012.02.020 22366604

[B18] Dong T. Knoshaug E. P. Davis R. Laurens L. M. L. Wychen S. V. Pienkos P. T. (2016). Combined algal processing: a novel integrated biorefinery process to produce algal biofuels and bioproducts. *Algal Res.* 19 316–323. 10.1016/j.algal.2015.12.021

[B19] Egerton S. Culloty S. Whooley J. Stanton C. Ross R. P. (2018). The gut microbiota of marine fish. *Front. Microbiol.* 9:873 10.3389/fmicb.2018.00873 PMC5946678 29780377

[B20] Gerde J. A. Wang T. Yao L. Jung S. Johnson L. A. Lamsal B. (2013). Optimizing protein isolation from defatted and non-defatted *Nannochloropsis* microalgae biomass. *Algal Res.* 2 145–153. 10.1016/j.algal.2013.02.001

[B21] Ghosh R. Mitra A. (2015). Suitability of green macroalgae *Enteromorpha intestinalis* as a feed form *Macrobrachium rosenbergii*. *J. Fish. Livest Prod.* 3 1–10.

[B22] Grammes F. Reveco F. E. Romarheim O. H. Landsverk T. Mydland L. T. Øverland M. (2013). *Candida utilis* and *Chlorella vulgaris* counteract intestinal inflammation in Atlantic Salmon (*Salmo salar* L.). *PLoS One* 8:e83213. 10.1371/journal.pone.0083213 24386162 PMC3873917

[B23] Haas S. Bauer J. L. Adakli A. Meyer S. Lippemeier S. Schwarz K. (2016). Marine microalgae *Pavlova viridis* and *Nannochloropsis* sp. *as n-*3 PUFA source in diets for juvenile European sea bass (*Dicentrarchus labrax* L.). *J. Appl. Phycol.* 28 1011–1021. 10.1007/s10811-015-0622-5

[B24] Herbert D. Phipps P. J. Strange R. E. (1971). “Chemical analysis of microbial cells,” in *Methods in microbiology*, eds Norris J. R. Ribbons D. W. (London: Academic press), 209–344. 10.1016/s0580-9517(08)70641-x

[B25] Jones A. C. Mead A. Kaiser M. J. Austen M. C. V. Adrian A. W. Auchterlonie N. A. (2014). Prioritization of knowledge needs for sustainable aquaculture: a national and global perspective. *Fish Fish.* 16 668–683.

[B26] Kiron V. Sørensen M. Huntley M. Vasanth G. K. Gong Y. Dahle D. (2016). Defatted biomass of the microalga, *Desmodesmus* sp., can replace fishmeal in the feeds for Atlantic salmon. *Front. Mar. Sci.* 3:67 10.3389/fmars.2016.00067

[B27] Laurens L. M. L. Nagle N. Davis R. Sweeney N. Van Wychen S. Lowell A. (2015). Acid catalyzed algal biomass pretreatment for integrated lipid and carbohydrate based biofuels production. *Green Chem.* 17 1145–1158. 10.1039/c4gc01612b

[B28] Lowe R. H. Evans H. J. (1964). Preparation and properties of a soluble nitrate reductase from *Rhizobium japonicum*. *Biochim. Biophys. Acta.* 85 377–389. 10.1016/0926-6569(64)90301-3 14194853

[B29] Mandal S. Patnaik R. Singh A. K. Mallick N. (2013). Comparative assessment of various lipid extraction protocols and optimization of transesterification process for microalgal biodiesel production. *Environ. Technol.* 34 2009–2018. 10.1080/09593330.2013.827730 24350454

[B30] Marinho G. S. Alvarado-Morales M. Angelidaki I. (2016). Valorization of macroalga *Saccharina latissima* as novel feedstock for fermentation-based succinic acid production in a biorefinery approach and economic aspects. *Algal Res.* 16 102–109. 10.1016/j.algal.2016.02.023

[B31] Mustafa M. G. Nakagawa H. (1995). A review: dietary benefits of algae as an additive in fish feed. *Isr. J. Aquacult. Bamid.* 47 155–162.

[B32] Nandeesha M. C. De Silva S. S. Krishnamoorthy D. Dathathri K. (1994). Use of mixed feeding schedules in fish culture: field trials on catla, *Catla catla* (Hamilton-Buchanan), rohu, *Labeo rohita* (Hamilton), and common carp, *Cyprinus carpio* L. *Aquacult. Fish Manage.* 25 659–670. 10.1111/j.1365-2109.1994.tb00730.x

[B33] Nandeesha M. C. Gangadhara B. Manissery J. K. Venkataraman L. V. (2001). Growth performance of two Indian major carps, catla (*Catla catla*) and rohu (*Labeo rohita*) fed diets containing different levels of *Spirulina platensis*. *Bioresour. Technol.* 80 117–120. 10.1016/s0960-8524(01)00085-2 11563701

[B34] Nelson D. L. Cox M. (2000). “Amino acid oxidation and the production of urea,” in *Lehninger Principles of Biochemistry*, eds Ryan M. Strange L. Neal V. (New York, NY: Worth Publishers), 643.

[B35] Neori A. (2011). “Green water” microalgae: the leading sector in world aquaculture. *J. Appl. Phycol.* 23 143–149. 10.1007/s10811-010-9531-9

[B36] Nicholas D. J. Nason A. (1957). “Determination of nitrate and nitrite,” in *Methods in Enzymology III*, eds Colowick S. P. Kaplan N. O. (New York, NY: Academic Press), 961–984.

[B37] Patnaik R. Mallick N. (2015). Utilization of *Scenedesmus obliquus* biomass as feedstock for biodiesel and other industrially important co-products: an integrated paradigm for microalgal biorefinery. *Algal Res.* 12 328–336. 10.1016/j.algal.2015.09.009

[B38] Radhakrishnan S. Saravana Bhavan P. Seenivasan C. Muralisankar T. (2015). Effect of dietary replacement of fishmeal with *Chlorella vulgaris* on growth performance, energy utilization and digestive enzymes in *Macrobrachium rosenbergii* postlarvae. *Int. J. Fish. Aquac.* 7 62–70.

[B39] Shah M. R. M. D. Lutzu G. A. Alam A. M. D. Sarker P. Chowdhury M. A. K. Parsaeimehr A. (2017). Microalgae in aquafeeds for a sustainable aquaculture industry. *J. Appl. Phycol* 30 197–213. 10.1007/s10811-017-1234-z

[B40] Siddhuraju P. Becker K. (2003). Comparative nutritional evaluation of differentially processed mucuna seeds (*Mucuna pruriens* (L.) *DC var. utilis (Wall ex Wight)* Baker ex Burck, on growth performance, feed utilization and body composition in *Nile Tilapia (Oreochromis niloticus L.)*. *Aquaculture* 34 487–500. 10.1046/j.1365-2109.2003.00836.x

[B41] Soeder C. J. Bolze A. (1981). Sulphate deficiency stimulates the release of dissolved organic matter in synchronus culture of *Scenedesmus obliquus*. *Plant Physiol.* 52 233–238. 10.1111/j.1399-3054.1981.tb08498.x

[B42] Sward A. (2014). *Muscle Mystery: Does Denatured Protein Still Make You Grow.* Available at: https://www.bodybuilding.com/fun/muscle-mystery-does-denatured-protein-still-make-you-grow.html (accessed January 14, 2017).

[B43] Teimouri M. Amirkolaie A. K. Yeganeh S. (2013). The effects of dietary supplement of *Spirulina platensis* on blood carotenoid concentration and fillet color stability in rainbow trout (*Oncorhynchus mykiss*). *Aquaculture* 414 224–228. 10.1016/j.aquaculture.2013.08.015

[B44] Vadstein O. Attramadal K. J. K. Bakke I. Forberg T. Olsen Y. Verdegem M. (2018). Managing the microbial community of marine fish larvae: a holistic perspective for larviculture. *Front. Microbiol.* 9:1820. 10.3389/fmicb.2018.01820 30210457 PMC6119882

[B45] Valente L. M. P. Gouveia A. Rema P. Matos J. Gomes E. F. Pinto I. S. (2006). Evaluation of three seaweeds *Gracilaria bursa-pastoris*, *Ulva rigida* and *Gracilaria cornea* as dietary ingredients in European sea bass (*Dicentrarchus labrax*) juveniles. *Aquaculture* 252 85–91. 10.1016/j.aquaculture.2005.11.052

[B46] Vestrum R. I. Attramadal K. J. K. Winge P. Li K. Olsen Y. Bones A. M. (2018). Rearing water treatment induces microbial selection influencing the microbiota and pathogen associated transcripts of cod (*Gadus morhua*) larvae. *Front. Microbiol.* 9:851. 10.3389/fmicb.2018.00851 29765364 PMC5938384

[B47] Wang W. Vignani R. Scali M. Cresti M. (2006). A universal and rapid protocol for protein extraction from recalcitrant plant tissues for proteomic analysis. *Electrophoresis.* 27 2782–2786. 10.1002/elps.200500722 16732618

[B48] Wessel D. Flugge U. I. (1984). A method for the quantitative recovery of protein in dilute solution in the presence of detergents and lipids. *Anal. Biochem.* 138 141–143. 10.1016/0003-2697(84)90782-6 6731838

